# Regulatory T-Cells Mediate IFN-α-Induced Resistance against Antigen-Induced Arthritis

**DOI:** 10.3389/fimmu.2018.00285

**Published:** 2018-02-19

**Authors:** Sudeep Chenna Narendra, Jaya Prakash Chalise, Sophie Biggs, Ulrich Kalinke, Mattias Magnusson

**Affiliations:** ^1^Division of Rheumatology, Autoimmunity and Immune Regulation, Department of Clinical and Experimental Medicine, Linköping University, Linköping, Sweden; ^2^Immune Regulation, IFReC, Osaka University, Suita, Japan; ^3^Twincore, Zentrum für Experimentelle und Klinische Infektionsforschung, Hannover, Germany

**Keywords:** interferon-alpha, regulatory T-cells, experimental arthritis, indoleamine 2,3-dioxygenase, kynurenine

## Abstract

**Objective:**

CD4^+^FoxP3^+^CD25^+^ regulatory T-cells (T_regs_) are important for preventing tissue destruction. Here, we investigate the role of T_regs_ for protection against experimental arthritis by IFN-α.

**Methods:**

Arthritis was triggered by intra-articular injection of methylated bovine serum albumin (mBSA) in wild-type mice, Foxp3DTReGFP^+/−^ mice [allowing selective depletion of T_regs_ by diphtheria toxin (DT)] and CD4-Cre^+/−^ IFNA1R flox/flox mice (devoid of IFNAR signaling in T-cells) earlier immunized with mBSA, with or without treatment with IFN-α or the indoleamine 2,3-dioxygenase (IDO)-metabolite kynurenine. T_regs_ were depleted in DT-treated Foxp3DTReGFP^+/−^ mice and enumerated by FoxP3 staining. Suppressive capacity of FACS-sorted CD25^+high^CD4^+^ T_regs_ was tested *in vivo* by adoptive transfer and *ex vivo* in cocultures with antigen-stimulated CFSE-stained T-responder (CD25^−^CD4^+^) cells. IDO was inhibited by 1-methyl tryptophan.

**Results:**

Both control mice and mice devoid of IFNAR-signaling in T helper cells were protected from arthritis by IFN-α. Depletion of T_regs_ in the arthritis phase, but not at immunization, abolished the protective effect of IFN-α and kynurenine against arthritis. IFN-α increased the number of T_regs_ in *ex vivo* cultures upon antigen recall stimulation but not in naïve cells. IFN-α also increased the suppressive capacity of T_regs_ against mBSA-induced T-responder cell proliferation *ex vivo* and against arthritis when adoptively transferred. The increased suppressive activity against proliferation conferred by IFN-α was clearly reduced by *in vivo* inhibition of IDO at immunization, which also abolished the protective effect of IFN-α against arthritis.

**Conclusion:**

By activating IDO during antigen sensitization, IFN-α activates T_regs_, which prevent arthritis triggered by antigen rechallenge. This is one way by which IFN-α suppresses inflammation.

## Introduction

Type I interferons are antiviral cytokines that also modulate inflammation including inflammatory diseases either in a pro-inflammatory or in an anti-inflammatory way (1). In systemic lupus erythematous and psoriasis, they are believed to mediate inflammation (2) whereas in diseases such as multiple sclerosis (3), colitis (4), and experimental arthritis (5–8) they may resolve inflammation. These apparently contradictory pro- and anti-inflammatory effects of type I IFNs are also reflected in their effects on regulatory T-cells (T_regs_). T_regs_, a distinct subset of FoxP3^+^, CD25^++^CD4^+^ T helper cells, are responsible for maintaining self-tolerance and suppressing aberrant immune responses during infection (9) but may also limit important antiviral and antineoplastic responses (10). Absence of strong innate co-stimulation during antigen presentation including, but not limited to, TGF-β (11), indoleamine 2,3-dioxygenase (IDO) (12) and aryl hydrocarbon receptor signaling (13) can promote peripheral T_reg_ development. The impact of type I IFN on T_reg_ development and function ranges from enhancing to direct inhibitory effects on T_regs_ (14). Direct inhibiton of T_regs_ by type I IFN can be found early during viral infections, which allows development of effective antiviral responses (15). Positive regulation of T_regs_ may be one of several anti-inflammatory mechanisms of type I IFN treatment against MS (16) and also a way by which tolerogenic dendritic cells mitigate inflammation (17). Defining the circumstances promoting either outcome will help controlling antimicrobial, antineoplastic, and autoimmune responses.

We have earlier shown that type I IFN (IFN-α) protects against antigen-induced arthritis (AIA) (6) and decreases antigen-specific proliferation and inhibits production of pro-inflammatory cytokines (IL-1β, IL-6, IL-17, TNF, IL-12, and IFN-gamma) while at the same time increasing the production of TGF-β (7) and the immune-modulatory enzyme IDO1, the latter two both crucial for the anti-inflammatory effect of IFN-α (8). In this study, we have thoroughly investigated the impact of IFN-α on T_regs_ quantitatively and qualitatively in AIA, which is a T cell-driven experimental model of arthritis with histopathological features resembling those found in RA (18). We show that T_regs_ mediate the anti-inflammatory effect of IFN-α during the arthritis phase of AIA and that IFN-α enhances the suppressive effect of T_regs_ in an IDO-dependent manner.

## Materials and Methods

### Mice

SV129 EV mice and *FoxP3DTReGFP* mice were originally from B and K Universal, North Humberside, England and Jackson Laboratories, ME, USA, respectively. *CD4-Cre^+/−^IFNAR flox/flox* and *CD4-Cre^−/−^IFNAR flox/flox* were received as a kind gift from Ulrich Kalinke, Twincore, Germany. Mice were further bred in the animal facility of Linkoping University, Sweden. Foxp3DTReGFP mice were bred heterozygously, and their offspring were genotyped for the mutant (*Foxp3DTReGFP^+/−^*) allele or wild-type (WT) allele (*Foxp3DTReGFP^−/−^*) by PCR using the primers 5′-CCCAGGTTACCATGGAGAGA-3′ and 5′-GAACTTCAGGGTCAGCTTGC-3′ for the mutant allele, and 5′-CAAATGTTGCTTGTCTGGTG-3′ and 5′-GTCAGTCGAGTGCACAGTTT-3′ (Invitrogen) for the WT allele according to the protocol for this strain provided by Jackson Laboratories at https://www.jax.org/strain/011003. Littermates without the mutant allele (WT) were used as control mice. All experimental procedures were performed according to the guidelines provided by the Swedish Animal Welfare Act and approved by the Ethical Committee Board, in Linköping (Dnr 12-01) and Stockholm (N271-14).

### Antigen-Induced Arthritis

**Figure 1:**
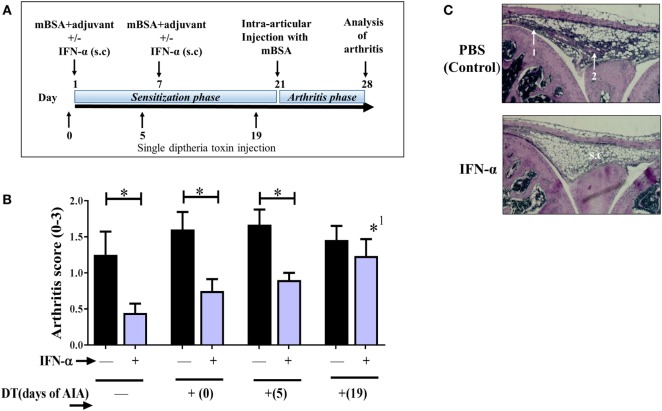
Regulatory T-cells (T_regs_) mediate the IFN-α-protection against antigen-induced arthritis (AIA). AIA was induced in female *Foxp3DTReGFP^+/−^* with or without 1,000 U IFN-α as described in Section “Materials and Methods.” Diphtheria toxin (DT) was administered i.p. as a single injection at day 0 or 5 or 19 of AIA for transient depletion of Foxp3^+^ T_regs_. **(A)** Graphical depiction of AIA and administration of DT. **(B)** The level of arthritis evaluated at day 28 of AIA is expressed as severity score (mean ± SEM, *n* ≥ 7). Comparison of arthritis severity score between different IFN-α-treated and -non-treated groups was done by the Mann–Whitney *U* test (*p < 0.05). *^1^Compared with no depletion in depletion of regulatory T-cells mice treated with IFN-α. **(C)**. Representative hematoxylin and eosin stained slides depicting arthritis severity score day 28 from control group (scored 2) and IFN-α-treated group (scored 0) from WT mice. White arrows indicate (1) thickening of synovial membrane, (2) synovial cell infiltration, and s.c denotes synovial cavity.

Arthritis was induced in female mice (8–13 weeks of age) as previously described (18) (Figure 1). Briefly, female mice were immunized subcutaneously with 200 µg methylated Bovine Serum Albumin (mBSA, Sigma-Aldrich) emulsified in Freund’s incomplete adjuvant (Sigma-Aldrich), with or without 1,000 U of IFN-α (IFNαA, PBL Assay Science, Piscataway, NJ, USA) or 15 mg/kg kynurenine (Sigma-Aldrich, St. Louise, MO, USA) also combined in the emulsion. One week later, a booster immunization of 100 µg mBSA emulsified in Freund’s incomplete adjuvant, with or without 1,000 U IFN-α or 15 mg/ml kynurenine, was injected at the base of the tail. On day 21, the mice were rechallenged with an intra-articular injection of 20 µl of mBSA solution (1.5 µg/µl) in the left knee joint. As a control, 20 µl of PBS was injected in the right knee joint. Mice were sacrificed on day 28, and knee joints were separated, fixed with 4% paraformaldehyde, decalcified and cut into sagittal sections (4 µm). Each section was stained with eosin and hematoxylin (Sigma-Aldrich) and the severity of arthritis was assessed with scores (0–3) on the basis of cellular infiltration in the synovial cavity, synovial layer thickening, and cartilage and bone erosion as earlier described (6). The score 0 represents no inflammation whereas score 1–3 represents increasing degrees of inflammation.

### T_reg_ Depletion *In Vivo*

Foxp3DTReGFP mice [also called depletion of regulatory T-cells (DEREG)] were utilized to deplete the T_regs_ *in vivo*. These mice carry a diphtheria toxin (DT) receptor gene coupled to a green fluorescent protein gene, which is controlled by a Foxp3 promoter (19). Upon administration of DT, the Foxp3^+^ T_reg_ population can be depleted for a limited period *in vivo* without affecting other cell populations. The T_regs_ repopulate to the original amount after 7–10 days (19). Following several optimization experiments, we finalized a single i.p. injection of 250 µg DT in 100 µl PBS that can deplete up to 90% of Foxp3^+^ T_regs_ 2 days after DT injection (Figure S1 in Supplementary Material). Foxp3^+^ T_regs_ were depleted in separate experimental groups each receiving a single injection of DT at either day 0, day 5, or day 19 of AIA.

### Cell Preparation and Immunostaining

Blood was collected from the tail vein on days 0, 4, 10, 14, 20, 24, and 28 from mice during AIA and mixed with heparin to prevent coagulation. Spleens and a pool of draining lymph nodes (axillary, popliteal, and inguinal) were collected on days 0, 4, 10, 24, and 28 of AIA, from which single cell suspensions were prepared by gently crushing and passing the spleen and lymph nodes through a 70 µm nylon cell strainer and lysing the red blood cells with an RBC lysing solution (Sigma-Aldrich, Dusseldorf, Germany). The single cell suspensions or 100 µl of heparinized blood were surface stained with rat anti-mouse CD4 FITC antibody (Clone GK1.5, BD Biosciences, San Jose, CA, USA) and rat anti-mouse CD25 PE antibody (Clone PC61.5, eBioscience, San Diego, CA, USA). The cells were then fixed and permeabilized with a Foxp3-staining set (eBioscience, USA) according to the manufacturer’s instructions and stained intra-cellularly with rat anti-mouse Foxp3 APC antibody (Clone FJK-16s, eBioscience, USA). Analysis was performed with FACS Gallios (Beckman Coulter, Inc., Brea, CA, United States), and collected data were analyzed with Kaluza^®^ Flow Analysis Software, Beckman Coulter (version 1.5). The percentages of Foxp3^+^ T_regs_ among gated CD4^+^ cells were determined by FMO gating as earlier described (20).

### *Ex Vivo* Restimulation of Leukocytes

A pooled single cell suspension of splenocytes and lymph node cells prepared as described above were re-suspended in Iscove’s Modified Dulbecco’s Media (Sigma-Aldrich) supplemented with 10% heat inactivated fetal bovine serum, 4 mM glutamine, 50 µM β-mercaptoethanol, 100 U/ml penicillin, and 0.1 mg/ml streptomycin (Sigma-Aldrich). 2 × 10^5^ cells of leukocytes were cultured for 72 h and stimulated with 50 µg/ml mBSA or 1 µg/ml anti-CD3 antibodies in the presence or absence of 500 U/ml of IFN-α. After 72 h, cells were harvested and analyzed for CD4, CD25, and Foxp3 expression as described above.

### *In Vivo* Treatment with 1-Methyl Tryptophan (1-MT)

Indoleamine 2,3-dioxygenase was blocked using DL 1-MT (Sigma-Aldrich), which was prepared in drinking water (5 mg/ml) as described previously (8). The 1-MT drinking solution was administered 2 days before the first immunization until day 20 of AIA.

### Suppression Assay

CD4^+^ T cells from single cell suspensions prepared from days 4, 10, 20, and 28 of AIA were sorted using magnetic bead based MACS technology (CD4^+^ T Cell Isolation Kit, Miltenyi Biotec, Cologne, Germany). From the sorted CD4^+^ cells, CD4^+^CD25^−^ cells, and CD4^+^CD25^+high^ cells were further sorted by FACS Aria using rat anti-mouse CD4 PE (Clone GK1.5, BioLegend, San Diego, CA, USA) and rat anti-mouse CD25 APC antibodies (Clone PC61, BioLegend, USA). For suppression assays, CD^+^CD25^−^ cells and CD4^+^CD25^+high^ cells were used as responder T cells (T_resp_ cells) and T_reg_, respectively. CFSE-stained T_resp_ cells from IFN-α-treated or -untreated mice at days 4, 10, 20, and 28 of AIA were prepared as described (21). 50,000 CFSE-stained T_resp_ cells isolated from IFN-α-treated or -non-treated mice were cocultured in a 96-well round-bottom culture plate with decreasing number of T_regs_ isolated from IFN-α-treated (with or without 1-MT in drinking water) or -non-treated mice in the T_reg_:T_resp_ ratio of 1:2, 1:4, 1:8, and 0:1 (T_resp_ cells only). In each well, 100,000 irradiated splenocytes as antigen presenting cells from naïve mice were added. Cultures were stimulated either with anti-CD3 (1 µg/ml) or mBSA (50 µg/ml). Cells were harvested after 72 h and analyzed by FACS Gallios, and the suppressive capacity of T_regs_ against T_resp_ cell proliferation was determined as previously described (22). The proliferation of CFSE-stained T cells was calculated based on CFSE MFI of proliferating cells and non-proliferating cells by Kaluza software. The suppression percentage was calculated as [(T_resp_ proliferation without T_regs_) − (T_resp_ proliferation with T_regs_)]/(T_resp_ proliferation without T_regs_) × 100.

### Adoptive Transfer of T_regs_

Spleens and draining lymph nodes (axillary, popliteal, and inguinal) were collected at day 20 of AIA from IFN-α-treated or -non-treated mice. CD4^+^CD25^+high^ T_regs_ were isolated from combined splenocytes and lymph node cell suspensions as described earlier. 50,000 or 250,000 T_regs_ in a volume of 100 µl PBS were intravenously injected in the tails of mice at day 20 of AIA. Arthritis was induced in these recipient mice by intra-articular injection of mBSA as described earlier on day 21 (1 day after transfer). The mice were sacrificed at day 28, and arthritis severity was evaluated as described earlier.

### Statistical Analysis

Differences in arthritis severity between groups with different treatments were measured by Mann–Whitney *U* test. Student’s *t*-test was used to compare the suppressive capacity of T_regs_ from PBS or IFN-α-treated mice (with or without *in vivo* 1-MT). A *p* value <0.05 was considered significant. All statistical tests were performed using GraphPad Prism 7.02.

## Results

### T_regs_ Mediate the IFN-α-Protection against AIA

Previously, we have shown that IFN-α protects against AIA (6, 8). Here, we wanted to determine if protection rendered by IFN-α in AIA is dependent on T_regs_. To this end we used DEREG mice where the Foxp3^+^ population can be transiently depleted *in vivo* by DT. DT was administered once at day 0, day 5, or day 19 during AIA resulting in a 90% depletion of Foxp3^+^ in blood 2 days after DT injection (Figure S1 in Supplementary Material). DT injection to wt littermate mice (*Foxp3DTReGFP^−/−^*) did not result in T_reg_ depletion (data not shown). The effect of T_reg_ depletion on arthritis in IFN-α-treated and control DEREG mice was evaluated on day 28 of AIA. In DEREG mice where the DT was administered at day 0 or 5 (sensitization phase of AIA), IFN-α could still significantly mitigate arthritis as compared with control DEREG mice receiving DT day 0 or day 5, respectively (Figure 1B). However, in DEREG mice where DT was administered at day 19 (i.e., shortly before arthritis induction by intra-articular injection of mBSA), the protective effect of IFN-α was totally abolished (Figure 1A), i.e., not significantly different compared with control DEREG mice receiving DT day 19 (Figure 1B) and significantly different from IFN-treated DEREG mice without depletion. This indicates that T_regs_ are critically required for IFN-α-protection in the arthritis phase of AIA (Figures 1B,C).

### IFN-α Treatment *In Vivo* Increases the *In Vitro* Suppressive Activity of T_regs_

**Figure 2:**
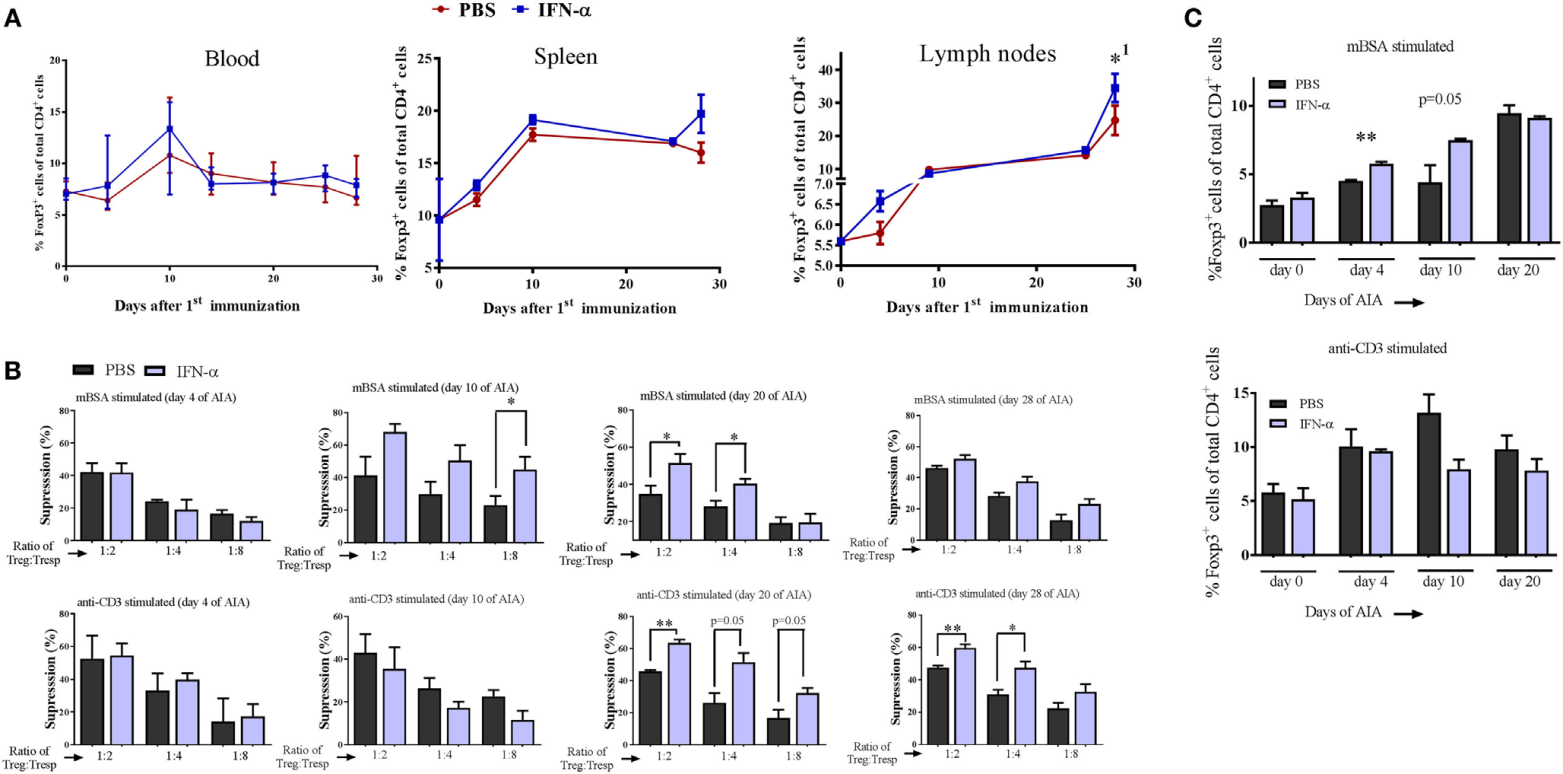
IFN-α treatment *in vivo* increases the *in vitro* suppressive activity of regulatory T-cells (T_regs_). **(A)** Percentage of Foxp3^+^ T_regs_ of gated CD4^+^ cells in blood, splenocytes, and lymph nodes collected at days 0, 4, 10, 24, and 28 during antigen-induced arthritis (AIA) from mice treated or not with IFN-α, *n* ≥ 5. *^1^Indicates that T_reg_ numbers in both groups were significantly higher day 28 as compared with day 0. **(B)** Percent suppression by T_regs_ (CD4^+^CD25^+high^) isolated from PBS or IFN-α-treated mice at days 4, 10, 20, and 28 against proliferation of T_resp_ cells (CD4^+^CD25^−^ from untreated mice) isolated the same days of AIA and stimulated with methylated bovine serum albumin (mBSA) (top) or anti-CD3 (bottom). Suppression against proliferation of T_resp_ cells by T_regs_ at decreasing T_reg_:T_resp_ cell ratios after 72 h culture was calculated as described in Section “Materials and Methods.” **(C)** (Top) Percentage Foxp3^+^ T_regs_ of gated CD4^+^ cells from spleen and lymph node cells restimulated *ex vivo* with mBSA. (Bottom) Percentage Foxp3^+^ T_regs_ of gated CD4^+^ cells from spleen and lymph node cells restimulated with anti-CD3. Spleens and lymph nodes were collected from mice subjected to AIA on days 0, 4, 10, and 20. Cells were prepared as described in Section “Materials and Methods” and restimulated *ex vivo* with mBSA or anti-CD3 for 72 h in presence or absence of IFN-α (500 U/ml). After 72 h, the cells were analyzed by FACS for T_regs_ quantification. Student’s *t*-test was used to compare differences between groups (*p < 0.05 and **p < 0.01; *n* = ≥ 5).

We analyzed the percentage of T_regs_ (Foxp3^+^ cells) among CD4^+^ cells in blood, spleens, and draining lymph nodes collected from IFN-α-treated and non-treated mice at different days during AIA. The percentage of Foxp3^+^ T_regs_ in IFN-α-treated mice did not differ significantly from percentages in non-treated mice in the blood at any day (Figure 2A). Likewise, no significant difference was observed in spleen or lymph nodes, although the total number of Foxp3^+^ T_regs_ increased in lymph nodes from day 0 to day 28 (Figure 2A). To test whether IFN-α affected the function of T_regs_, we performed T_reg_ suppression assays. The *ex vivo* suppressive capacity of T_regs_ (CD4^+^CD25^+high^) isolated at days 4, 10, 20, and 28 of AIA from PBS (control) or IFN-α-treated mice was tested against proliferation of T-responder cells (T_resp_ cells, CD4^+^CD25^−^), isolated at the same days of AIA either from control mice, or from IFN-α-treated mice. For the suppressive assay, 50,000 CFSE-stained T_resp_ cells were cocultured with APCs and decreasing numbers of T_regs_ (T_reg_:T_resp_ ratios 1:1, 1:2, 1:4, and 1:8) and stimulated with mBSA or anti-CD3 for 72 h. The suppressive capacity of T_regs_ was thereafter evaluated by flow cytometry by comparing the proliferation of T_resp_ cells in cocultures relative to T_resp_ cells without T_regs_. As depicted in Figure 2B, the more T_regs_ that are present in the cultures, the higher the suppression (see, e.g., top panel in Figure 2B: T_reg_:T_resp_ ratio 1:2 vs T_reg_:T_resp_ ratio 1:4 or 1:8). No significant differences were observed in the suppressive capacity of T_regs_ isolated at day 4 between PBS and IFN-α-treated mice (Figure 2B, day 4). T_regs_ isolated at day 10 and day 20 from IFN-α-treated mice during AIA exhibited clearly higher suppression of antigen-specific (mBSA)-induced proliferation than T_regs_ from PBS-treated mice. From day 20 and onward, an enhanced suppressive capacity of T_regs_ isolated from IFN-α-treated mice was in fact also observed against polyclonal (anti-CD3) stimulation of T_resp_ cells (Figure 2B, day 20 and day 28). The increased suppressive capacity of T_regs_ from IFN-α-treated mice was also observed on proliferation of T_resp_ cells isolated from IFN-α-treated mice (Figure S2 in Supplementary Material).

Three days after the second immunization (day 10) we observed an increased suppressive capacity of T_regs_ from IFN-α-treated mice (Figure 2B, day 10). This was observed when T_resp_ cells were restimulated with the immunizing antigen (mBSA), but not when the same T_resp_ cells were stimulated with the polyclonal activator anti-CD3 (Figure 2B, day 10). To explore this antigen requirement further, we counted the number of T_regs_ generated *in vitro* by IFN-α in the presence or absence of antigen-specific restimulation. Pooled spleen and lymph node cells, either from naïve mice or from mice immunized once or twice with the antigen (mBSA) were restimulated for 72 h with mBSA or anti-CD3, with or without 500 U of IFN-α. No effect of the mBSA antigen or IFN-α on the quantity of T_regs_ was observed in naïve leukocytes when stimulated with anti-CD3 or mBSA in the presence of IFN-α (Figure 2C). In contrast to naïve cell cultures, FoxP3^+^CD4^+^ T_regs_ were significantly increased by IFN-α in cultures using cells from mice once or twice immunized with mBSA, when restimulated *ex vivo* with mBSA + IFN-α (Figure 2C). The IFN-α-mediated increase of T_regs_ was not observed when cells from mBSA-immunized mice were stimulated with anti-CD3 antibodies plus IFN-α (Figure 2C). Thus, IFN-α increases the number of T_regs_ *in vitro*, but only in the presence of antigen in already antigen-sensitized cells.

### Type I IFN Signaling in T Helper Cells Is Not Required for IFN-α-Protection against AIA

**Figure 3:**
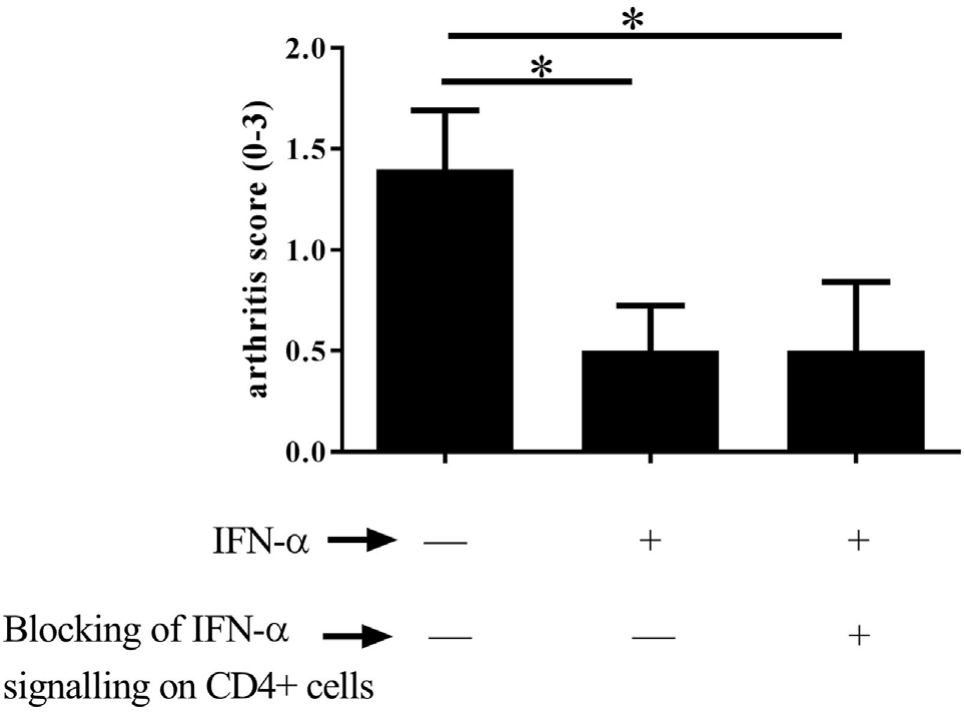
Type I IFN signaling in T helper cells is not required for IFN-α-protection against antigen-induced arthritis (AIA). AIA was induced in female CD4-Cre^+/−^ IFNAR flox/flox mice or their wild-type littermates CD4-Cre^−/−^ IFNAR flox/flox mice with or without 1,000 U IFN-α as described in Section “Materials and Methods.” The level of arthritis evaluated at day 28 of AIA is expressed as severity score (mean ± SEM, *n* ≥ 7). Comparison of arthritis severity score between different IFN-α-treated and -non-treated groups was done by the Mann–Whitney *U* test (*p < 0.05).

As T_regs_ are crucial for protection against arthritis by IFN-α, we investigated whether protection is a direct effect of IFN-α on T helper cells. To this end the protective effect of IFN-α against AIA was tested in CD4-Cre^+/−^ IFNAR flox/flox mice where the IFN-α receptor (type I IFN receptor) is knocked out in T cells (23). When treated with IFN-α, mice lacking the type I IFN receptor on CD4^+^ T cells were equally protected against AIA by IFN-α compared with their WT counterpart (CD4-Cre^−/−^ IFNAR flox/flox), as depicted in (Figure 3).

### Enzymatic IDO Activity Mediates the Increased Suppressive Capacity of T_regs_ Conferred by IFN-α

**Figure 4:**
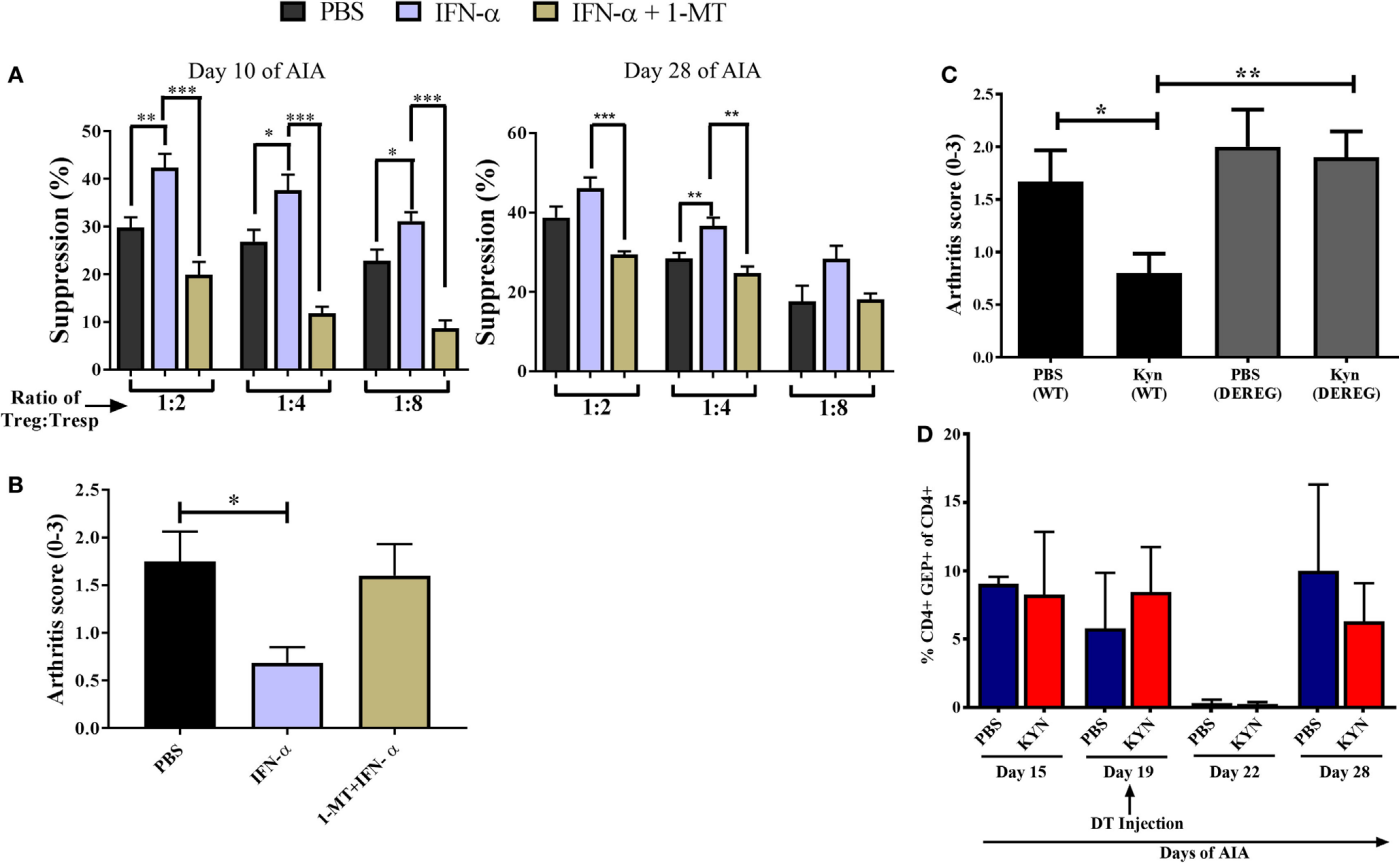
Enzymatic indoleamine 2,3-dioxygenase activity mediates the increased suppressive capacity of regulatory T-cells (T_regs_) conferred by IFN-α. T_regs_ (CD4^+^CD25^+high^) from mice treated with IFN-α with or without 1-methyl tryptophan (1-MT) treatment [during sensitization phase as described in Ref. (8)] and T_resp_ cells (CD4^+^CD25^−^) from non-treated mice was isolated at days 10 and 28 of antigen-induced arthritis (AIA). **(A)** Percent suppression by T_regs_ isolated from PBS, IFN-α, and IFN-α + 1-MT-treated mice at days 10 and 28 against proliferation of T_resp_ cells (from untreated mice) isolated the same days of AIA and stimulated with methylated bovine serum albumin. Student’s *t*-test was used to compare suppressive capacity between groups (*p < 0.05, **p < 0.01, and ***p < 0.001). **(B)** The level of arthritis in untreated mice or mice treated with IFN-α with or without 1-MT treatment [during sensitization phase as described in Ref. (8)] evaluated at day 28 of AIA expressed as severity score (mean ± SEM, *n* ≥ 5). **(C)** The level of arthritis in *Foxp3DTReGFP^+/−^* or their wild-type littermates *Foxp3DTReGFP^−/−^* treated or not with KYN as described (8). All mice in panel **(C)** were injected with diphtheria toxin (DT) intra-peritoneally on day 19 during the course of AIA. The level of arthritis evaluated at day 28 of AIA is expressed as severity score (mean ± SEM, *n* ≥ 4). Comparison of arthritis severity score between different groups in panels **(B,C)** were done by the Mann–Whitney *U* test (*p < 0.05) (*n* ≥ 4). **(D)** Percentage of CD4^+^GFP^+^ cells in DEREG mice before, during, and after depletion phase of T_regs_. The data shown are mean values ± SEM of each group (*n* ≥ 4).

Because IDO is implicated in the generation of T_regs_ (24, 25) we tested whether IDO contributed to the increased suppressive function conferred by IFN-α on T_regs_ during AIA. To this end, we performed the same *ex vivo* suppressive assay with T_regs_ from IFN-α-treated mice as described in Figure 2B, but in which the IDO inhibitor 1-MT was administered during the sensitization phase of AIA as earlier described in Ref. (8). Presence of 1-MT during Ag sensitization did not impair the suppressive function of T_regs_ in general, but the enhancing effect of IFN-α on the suppressive capacity of T_regs_ (Figure 2B) was clearly hampered if mice were treated with the IDO inhibitor 1-MT (Figure 4A). This was apparent both in T_regs_ isolated in the sensitization phase (day 10 of AIA) as well as in T_regs_ isolated after induction of arthritis (day 28 of AIA). In line with the abolished increased suppressive capacity of T_regs_, IFN-α + 1-MT-treated mice were not protected against arthritis as were mice receiving IFN-α + vehicle (Figure 4B) and as earlier reported (8).

Thus, one way by which IFN confers a better suppressive capacity upon T_regs_ is by activating the enzymatic activity of IDO, which converts tryptophan to kynurenine. To test whether kynurenine also employs T_regs_ to prevent arthritis, Kyn was administered at immunization in AIA to DEREG mice with or without depletion of T_regs_ by DT. As depicted in Figure 4C, Kyn readily protected DEREG mice from arthritis, in line with our earlier observations (8). When mice were treated with DT on day 19, resulting in a potent but transient depletion of Foxp3^+^ T_regs_ (Figure 4D), the protective effect was abolished (Figure 4C). Thus, as for IFN-α (Figure 1A) T_regs_ are required for the protective effect of Kyn against AIA.

### Adoptive Transfer of T_regs_ from IFN-α-Treated Mice Protects Against mBSA Induced Arthritis

**Figure 5:**
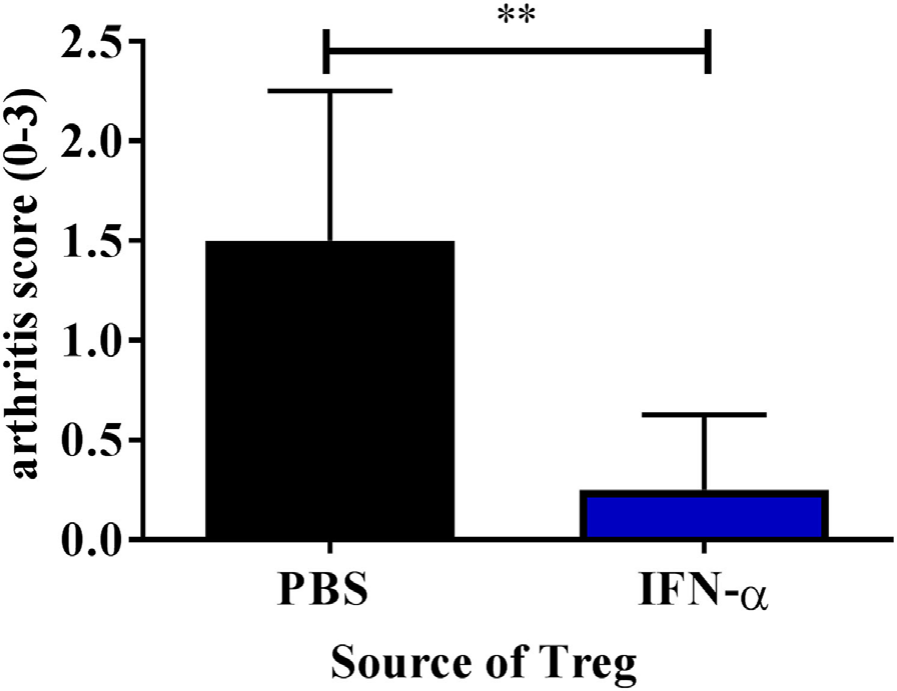
Adoptive transfer of T_regs_ from IFN-α-treated mice protects against mBSA induced arthritis. Spleens and draining lymph nodes from PBS and IFN-α-treated mice undergoing antigen-induced arthritis (AIA) were collected at day 20 of AIA. 50,000 T_regs_ (CD4^+^CD25^+high^) were sorted using FACS and were injected intravenously in presensitized recipient mice on day 20. These recipient mice were subjected to arthritis induction on day 21. The level of arthritis evaluated at day 28 of AIA is expressed as severity score (mean ± SEM, *n* ≥ 4). Comparison of arthritis severity score between presensitized recipient mice receiving either T_regs_ from PBS or IFN-α-treated mice was done by the Mann–Whitney *U* test (*p < 0.05 and **p < 0.01; *n* = 6).

Knowing that IFN-α treatment *in vivo* can enhance the *ex vivo* suppressive activity of T_regs_, we tested the ability of such T_regs_ to protect against AIA. To this end we isolated T_regs_ from mice immunized with mBSA with or without IFN-α (20 days after the first immunization). 50,000 FACS- sorted T_regs_ (CD4^+^CD25^+high^) were intravenously transferred to recipient mice subjected to AIA (day 20 of AIA). Arthritis was induced in recipient mice one day after transfer (day 21) by intra-articular injection of mBSA, and the severity of arthritis was evaluated at day 28. Transfer of 50,000 T_regs_ from IFN-α-treated mice clearly prevented development of arthritis whereas the same number of T_regs_ from PBS-treated mice did not (Figure 5). To verify that T_regs_ from PBS-treated mice were still functional we transferred a five times higher dose of T_regs_ to mice subjected to AIA. Adoptive transfer of 250,000 T_regs_ from mBSA-immunized mice, irrespectively of whether IFN-α was included at immunization or not, to recipient mice subjected to AIA prevented arthritis development (data not shown). Thus, IFN-α confers a suppressive capacity to T_regs_, so that an inferior number of T_regs_ are sufficient to prevent arthritis development as compared with T_regs_ generated in the absence of IFN-α.

## Discussion

In this work, we describe the requirement of T_regs_ for the ability of IFN-α to protect against AIA and an enhancing effect of IFN-α on the suppressive capacity of T_regs_, both *in vivo* and *in vitro*.

For IFN-α to protect against AIA it must be present at the time of antigen sensitization (6). During sensitization, i.e., before inflammation is triggered by antigen reexposure in the joint, IFN-α activates the enzymatic activity of IDO (8) and production of TGF-β (7), which are both essential components of how IFN-α protects against AIA (8). Intriguingly, although both IDO and TGF-β are clearly required for the anti-inflammatory effect of IFN-α, they are both redundant for inhibition of inflammation once arthritis is triggered by antigen reexposure in the joint (8). By contrast, we here demonstrate that T_regs_ are crucial to prevent inflammation after antigen reexposure in IFN-α-treated mice because depletion of the Foxp3-expressing cells at this time point totally abolished the protective effect of IFN-α (Figure 1). Also, adoptive transfer at the onset of the arthritis phase of T_regs_ isolated from immunized mice potently prevented development of arthritis (Figure 5). Likewise, T_regs_ from mice protected against CIA by the anti-inflammatory environment conferred by pregnancy, not only protected against arthritis, but could also protect non-pregnant mice from CIA upon adoptive transfer (26).

Despite the protective effect played by T_regs_, we did not observe a significant increase in T_regs_ in mice treated with IFN-α but T_regs_ isolated from IFN-α treated, mBSA-immunized mice were at least four times more efficient in protecting against AIA than T_regs_ isolated from mBSA-immunized mice (Figure 5 and data not shown). This indicates that IFN-α increases the suppressive capacity of T_regs_. This was further confirmed *ex vivo* by isolation of T_regs_ from immunized mice, treated or not with IFN-α at immunizations. Already 3 days after the last mBSA-immunization, T_regs_ isolated from mice treated with IFN-α were significantly more suppressive than T_regs_ from control mice against T-cell proliferation elicited by mBSA-restimulation *ex vivo* (Figure 2).

The effects of IFN-α on the generation and suppressive capacity of T_regs_ are far from clear cut (14). Many observations suggest an inhibitory effect of IFN-α on the function of T_regs_ whereas others (27), like this study, describe enhancing effects of IFN-α on T_regs_.

In an EAE study similar to ours, Wang et al. immunized mice with MOG + IFN-β, which prevented encephalitis by generation of MOG-specific T_regs_ (28). The protective effect of IFN-β was clearly antigen-specific because protection by IFN-β required that the same antigens were used to tolerize and to elicit EAE. In the present study, the initial enhanced suppressive effect of IFN-α on T_regs_ (isolated from mice 3 days after the last immunization, day 10 of AIA) was only apparent when T-responder cells were restimulated with the same antigen (mBSA) and not by polyclonal stimuli (Figure 2B, day 10), also indicating antigen specificity in the development of suppressive capacity of T_regs_ induced by IFN-α.

Although we did not find an IFN-α-induced increase in T_regs_ *in vivo* during AIA, IFN-α increased the number of T_regs_ in antigen-recall stimulation cultures *ex vivo* (Figure 2C). Like this *in vitro* study (Figure 2C), Wang et al. also observed an increase in FoxP3^+^ T_regs_ *in vitro* by type I IFN during antigen re-stimulation (28). If the enhancing effect of IFN-α + mBSA on T_regs_ in AIA is antigen specific, an increase in mBSA-specific T-cells among total CD4^+^FoxP3 cells may be too low to detect as an increase in CD4^+^FoxP3^+^ cells *in vivo* (Figure 2A). In line with this, we could only detect an IFN-α-mediated increase of Foxp3^+^ T_regs_ during antigen recall stimulation *ex vivo* (Figure 2C). However, by using transgenic mice where the majority of T-cells are MOG-specific, Wang et al. could confirm an IFN-β driven, antigen-specific increase in FoxP3^+^ T_regs_ in animals protected by IFN-β (28). By contrast, during polyclonal activation of T-cells, type I IFN may prevent the expansion of T_regs_ (29), and we observed a similar effect *in vitro* using the polyclonal activator anti-CD3 (Figure 2C, lower panel). The inhibitory effect of IFN-α on T_regs_ generated during polyclonal activation may be due to a general inhibition of IL-2 from conventional T-cells that limit T_reg_ survival (30). Taken together, type I IFN may have different effects on polyclonally-driven vs antigen-specific T_reg_ proliferation, with an enhancing effect on the latter. It may, however, not be that clear cut because at later time points during AIA, we observed that T_regs_ from mice treated with mBSA + IFN-α also had an enhanced suppressive effect on T cells from mBSA-immunized mice re-stimulated with anti-CD3 (Figure 2B, days 20–28), indicating that the suppressive capacity of T_regs_ generated in the presence of IFN-α may also suppress polyclonally activated T-cells. Therefore, cautiousness in interpreting how IFN-α affects the suppressive capacity of T_regs_ is needed and further studies, including TCR transgenic mice, are required to explore the effect of IFN-α on T_reg_-mediated suppression of antigen-specific vs polyclonally activated T cell proliferation.

Direct vs indirect actions of type I IFN on T_regs_ *via* IFNAR may also shed light on the complex effects of type I IFN on T_regs_. In a model of acute viral infection, type I IFN has a direct but transient inhibitory effect on T_regs_ not observed in mice devoid of IFNAR expression on T_regs_ (15). By contrast, in the arthritis model presented here, the T_reg_-dependent protection against AIA conferred by IFN-α does not require the presence of IFNAR on T-cells (Figure 3). However, a direct effect, i.e., signaling *via* IFNAR on T_regs_, was in fact necessary for IFN-α-induced Foxp3 expression and suppressive function of T_regs_ in experimental IBD (31), and the inhibitory function of IFN-α on T_regs_ described by Pace et al. cited above was partly mediated *via* IFNAR-expressing APC (29). Thus, the contradictory effects of IFN-α on T_regs_ may not be explained by direct vs indirect effects of IFN-α. In fact, IFNAR expression on T_regs_ is an important regulator of their number and function and both enhancing (32) and limiting (15) direct effects of IFN-α have been reported.

We earlier identified pDC as a critical cell population for the protective effect of IFN-α against arthritis. Upon stimulation with IFN-α, pDC produce the enzyme IDO (8), which converts tryptophan to kynurenine, a critical component for the anti-proliferative effect of IFN-α in AIA (8). We here demonstrate that T_regs_ further mediate the protective effect of kynurenine against arthritis (Figure 4). In line with this, the enzymatic activity of IDO was required for the increased anti-proliferative effect of T_regs_ conferred by IFN-α (Figure 4). We therefore believe that IDO-producing pDC are the cells inducing suppressive T_regs_ in mice treated with IFN-α during AIA. Likewise, IDO-producing pDC activate T_regs_, which can suppress the development of EAE (33).

In conclusion, we here demonstrate that antigen sensitization in the presence of type I IFN promotes development of T_regs_ with ability to suppress inflammation triggered by the same antigen. This will help to create tolerance-based therapies to combat autoimmune diseases and allergy.

## Ethics Statement

All experimental procedures were done according to the guidelines provided by the Swedish Animal Welfare Act and approved by the Ethical Committee Board, in Linköping (Dnr 12-01) and Stockholm (N271-14).

## Author Contributions

Conceived and designed the experiments: SN, JC, UK, and MM. Performed the experiments: SN, JC, SB, and MM. Analyzed the data and wrote the paper: SN, JC, SB, UK, and MM. Contributed reagents/materials/analysis tools: UK and MM.

## Conflict of Interest Statement

The authors declare that the research was conducted in the absence of any commercial or financial relationships that could be construed as a potential conflict of interest.

## Supplementary Material

Figure S1 Diphtheria toxin (DT) was administered i.p. in *Foxp3DTReGFP^+/−^* mice for transient depletion of Foxp3^+^ regulatory T-cells (T_regs_). Cells were analyzed for CD4, FoxP3, as well as GFP expression in the blood before and after 3 days of DT injection. Percentage of CD4^+^GFP^+^ cells in DEREG mice before and after 3 days of DT injection.

Figure S2 *In vivo* IFN-α treatment enhances *in vitro* suppressive capacity of regulatory T-cells (T_regs_) against proliferation of T_resp_ cells from IFN-α-treated mice during antigen-induced arthritis (AIA). AIA was proceeded in female wild-type mice with or without 1,000 U IFN-α as described in Section “Materials and Methods.” T_regs_ (CD4^+^CD25^+high^) from IFN-α-treated or -non-treated mice and T_resp_ cells (CD4^+^CD25^−^) from IFN-α-treated mice was isolated at days 4, 10, 20, and 28 of AIA and suppression assay was run. Suppression against proliferation of T_resp_ cells by T_regs_ at decreasing T_reg_:T_resp_ cell ratios after 72 h culture in the presence of methylated bovine serum albumin (mBSA) or anti-CD3 was calculated as described in Section “Materials and Methods.” Percent suppression by T_regs_ isolated from PBS or IFN-α-treated mice at day 4, day 10, day 20, and day 28 against proliferation of T_resp_ cells (from IFNα-treated mice) isolated from same days of AIA and stimulated with mBSA (top) or anti-CD3 (bottom).
