# Supplementary figures and images for: Regulatory T-Cells Mediate IFN-α-Induced Resistance against Antigen-Induced Arthritis
Source: Front Immunol. 2018 Feb 19;9:285. doi: 10.3389/fimmu.2018.00285 (PMC5826073; doi:10.3389/fimmu.2018.00285)

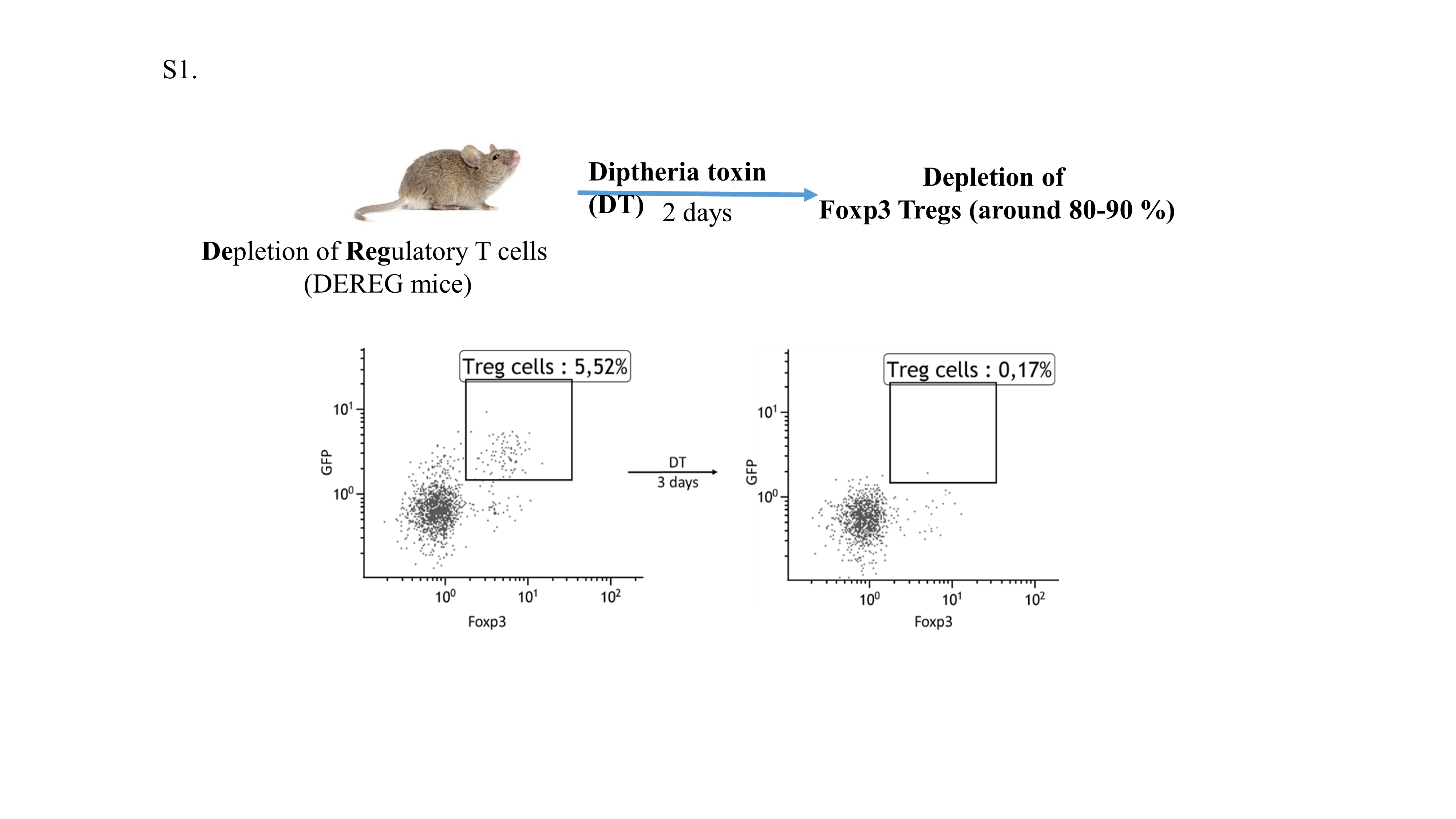

Supplement: Figure S1 — Diphtheria toxin (DT) was administered i.p. in Foxp3DTReGFP+/− mice for transient depletion of Foxp3+ regulatory T-cells (Tregs). Cells were analyzed for CD4, FoxP3, as well as GFP expression in the blood before and after 3 days of DT injection. Percentage of CD4+GFP+ cells in DEREG mice before and after 3 days of DT injection. [file Image_1.tif]

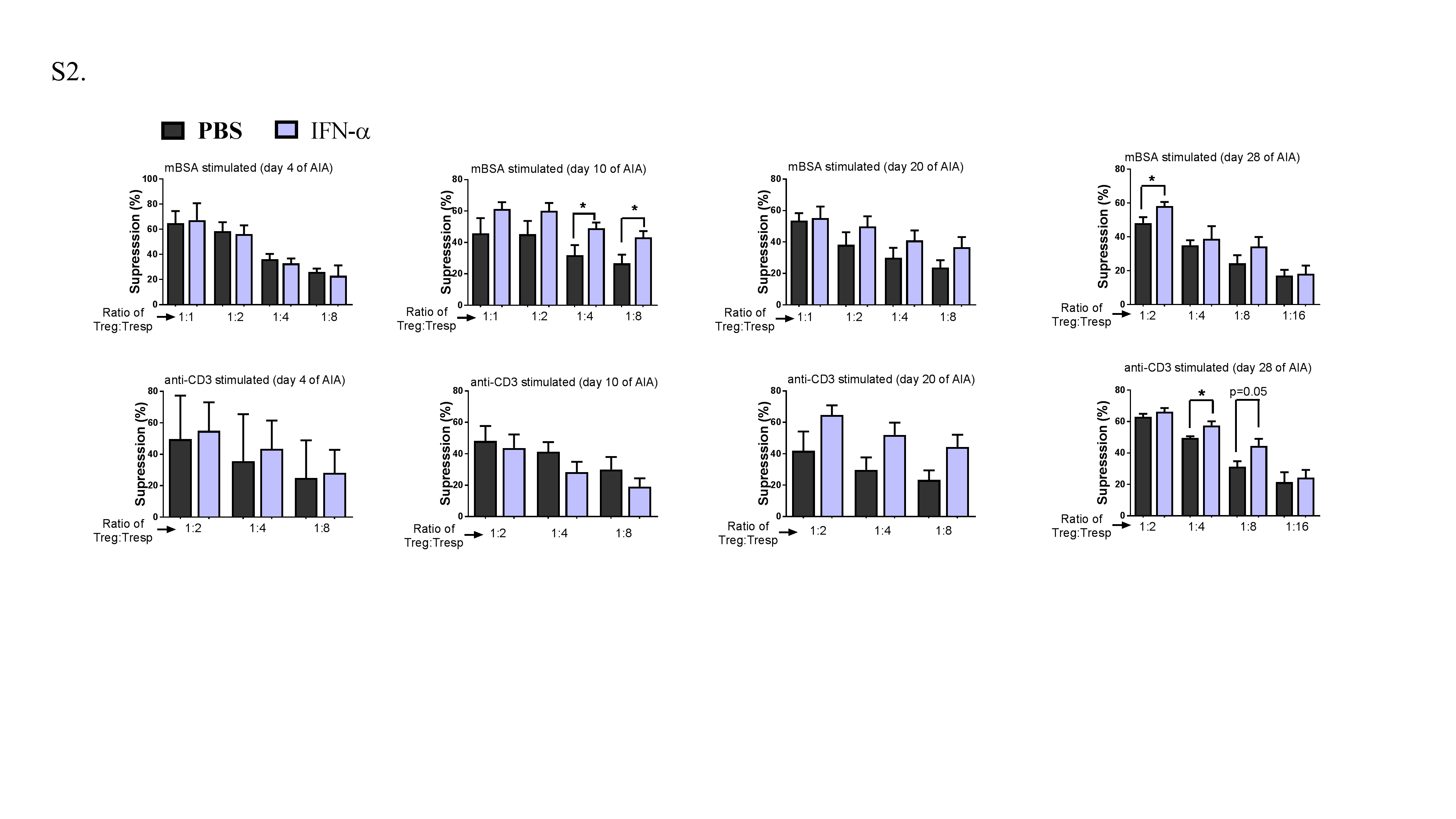

Supplement: Figure S2 — In vivo IFN-α treatment enhances in vitro suppressive capacity of regulatory T-cells (Tregs) against proliferation of Tresp cells from IFN-α-treated mice during antigen-induced arthritis (AIA). AIA was proceeded in female wild-type mice with or without 1,000 U IFN-α as described in Section “Materials and Methods.” Tregs (CD4+CD25+high) from IFN-α-treated or -non-treated mice and Tresp cells (CD4+CD25−) from IFN-α-treated mice was isolated at days 4, 10, 20, and 28 of AIA and suppression assay was run. Suppression against proliferation of Tresp cells by Tregs at decreasing Treg:Tresp cell ratios after 72 h culture in the presence of methylated bovine serum albumin (mBSA) or anti-CD3 was calculated as described in Section “Materials and Methods.” Percent suppression by Tregs isolated from PBS or IFN-α-treated mice at day 4, day 10, day 20, and day 28 against proliferation of Tresp cells (from IFNα-treated mice) isolated from same days of AIA and stimulated with mBSA (top) or anti-CD3 (bottom). [file Image_2.tif]
